# Cisatracurium stimulates testosterone synthesis in rat and mouse Leydig cells via nicotinic acetylcholine receptor

**DOI:** 10.1111/jcmm.16029

**Published:** 2020-10-27

**Authors:** Chaobo Ni, Yang Li, Zengqiang Li, Lili Tian, Jie Fu, Keyang Wu, Yiyan Wang, Ming Yao, Ren‐shan Ge

**Affiliations:** ^1^ Department of Anesthesiology Second Affiliated Hospital & Yuying Children's Hospital of Wenzhou Medical University Wenzhou China; ^2^ Department of Anesthesiology and Pain Research Center Jiaxing University Affiliated Hospital The First Hospital of Jiaxing Jiaxing China

**Keywords:** adult Leydig cells, CHRNA4, cisatracurium, MLTC‐1 cells, nAChR, testosterone

## Abstract

As a cis‐acting non‐depolarizing neuromuscular blocker through a nicotinic acetylcholine receptor (nAChR), cisatracurium (CAC) is widely used in anaesthesia and intensive care units. nAChR may be present on Leydig cells to mediate the action of CAC. Here, by Western blotting, immunohistochemistry and immunofluorescence, we identified that CHRNA4 (a subunit of nAChR) exists only on rat adult Leydig cells. We studied the effect of CAC on the synthesis of testosterone in rat adult Leydig cells and mouse MLTC‐1 tumour cells. Rat Leydig cells and MLTC‐1 cells were treated with CAC (5, 10 and 50 μmol/L) or nAChR agonists (50 μmol/L nicotine or 50 μmol/L lobeline) for 12 hours, respectively. We found that CAC significantly increased testosterone output in rat Leydig cells and mouse MLTC‐1 cells at 5 μmol/L and higher concentrations. However, nicotine and lobeline inhibited testosterone synthesis. CAC increased intracellular cAMP levels, and nicotine and lobeline reversed this change in rat Leydig cells. CAC may increase testosterone synthesis in rat Leydig cells and mouse MLTC‐1 cells by up‐regulating the expression of *Lhcgr* and *Star*. Up‐regulation of *Scarb1* and *Hsd3b1* expression by CAC was also observed in rat Leydig cells. In addition to cAMP signal transduction, CAC can induce ERK1/2 phosphorylation in rat Leydig cells. In conclusion, CAC binds to nAChR on Leydig cells, and activates cAMP and ERK1/2 phosphorylation, thereby up‐regulating the expression of key genes and proteins in the steroidogenic cascade, resulting in increased testosterone synthesis in Leydig cells.

## 1 INTRODUCTION

In addition to the hypothalamic‐pituitary‐gonadal axis, the nervous system may play a key role in Leydig cell function. Previous studies have shown that some nerve fibres reach the rat and human testes and may have an effect on Leydig cells.^1^, ^2^ The destruction of testicular innervation led to Leydig cell apoptosis and impaired Leydig cell function.^1^ The nicotinic acetylcholine receptor (nAChR) is a transmembrane ion channel that contains five subunits α2βγδ and belongs to the ligand‐gated receptor family.^3^ It is primarily distributed in the ganglion cell membrane, adrenal medulla, musculoskeletal neuromuscular junction and brain.^4^ The receptor may also be present in testicular cells because gene transcriptome analysis found that nAChR subunit α4 (*Chrna4*) is highly expressed in adult Leydig cells (ALCs) of rats.^5^

Non‐depolarizing muscle drugs (NMBDs) can competitively bind to nAChR without depolarizing the receptor, which can prevent acetylcholine from binding to nAChR, thereby relaxing skeletal muscle.^6^ One of the NMBDs is cisatracurium (CAC).^7^ CAC is the 1R cis‐1′R cis isomer of atracurium.^7^ CAC is widely used in clinic. It is an auxiliary method of general anaesthesia, used for rapid intubation and skeletal muscle relaxation during anaesthesia induction and surgery.^7^ In the intensive care unit, CAC is used to promote mechanical ventilation through sedation. It can also promote mechanical ventilation in patients with acute lung injury or acute respiratory distress syndrome.^8^, ^9^ Compared with other neuromuscular blocking drugs, CAC is more effective and does not produce cardiovascular effects or affect plasma histamine concentration after high‐dose administration.^10^ Although CAC is a nAChR antagonist, its effects on Leydig cell function remains unknown.

Previous study has shown that NMBDs may affect the male reproductive system. Rocuronium, one of the NMBDs, can cause testicular degeneration and inhibit spermatogenesis.^11^ Hexamethonium, another NMBD, can partially reverse the inhibitory effect of nicotine on testosterone production in ALCs of rats, suggesting that CAC may have an action on Leydig cells.^12^

The synthesis of testosterone in Leydig cells requires a complex chemical reaction chain.^13^, ^14^ First, Leydig cells need the starting material cholesterol. The cholesterol in Leydig cells is mainly absorbed from the circulatory of high‐density lipoprotein after its binding to the scavenger receptor class B member 1 (SCARB1) on the membrane of Leydig cells.^15^ Then, cholesterol is needed acutely transported to the inner membrane of the mitochondrion, which is achieved by steroidogenic acute regulatory protein (STAR) together with benzodiazepine receptor (TSPO).^16^, ^17^ Third, the first‐step steroidogenic reaction, catalysed by cholesterol side‐chain cleavage enzyme (CYP11A1) in the inner membrane of the mitochondrion, converts cholesterol to intermediate steroid 22R‐hydroxycholesterol and then cleaves the double bond of 22R‐hydroxycholesterol to generate pregnenolone. Finally, a series of enzymatic chain reactions happen in the smooth endoplasmic reticulum, with 3β‐hydroxysteroid dehydrogenase/Δ5→Δ4 isomerase (3β‐HSD1) catalysing pregnenolone to progesterone, 17α‐hydroxylase/17,20‐lyase (CYP17A1) catalysing progesterone to 17α‐hydroxyprogesterone and further to androstenedione, and 17β‐hydroxysteroid dehydrogenase isoform 3 (17β‐HSD3) catalysing androstenedione into testosterone.^18^ The testosterone synthesis requires the pulse regulation of pituitary‐secreted luteinizing hormone (LH), which binds to Leydig cell membrane receptor (LHCGR), a G‐coupled protein, to activate adenylate cyclase and increase intracellular cAMP levels for signalling.^19^ Here, we report the effects of CAC on these steps in rat and mouse Leydig cells, primary rat ALCs and mouse MLTC‐1 tumour Leydig cell line.

## 2 MATERIALS AND METHODS

### 2.1 Chemicals and animals

Cisatracurium was obtained from Jiangsu Hengrui Pharmaceutical Company. Nicotine was purchased from Sigma‐Aldrich. 22‐Hydroxycholesterol, pregnenolone, progesterone and androstenedione were purchased from Steraloids. 8Br‐cAMP and lobeline were purchased from Selleck. LH was provided by NIH. Primers of rat genes for real‐time quantitative PCR (qPCR) were listed in Table S1. Primers of mouse genes for qPCR were listed in Table S2. Antibodies for immunohistochemistry, immunofluorescence and Western blotting were listed in Table S3. Male Sprague‐Dawley rats (90 days old) were obtained from Shanghai Experimental Animal Center. All animal procedures for this experiment have been approved in accordance with the Institutional Animal Care and Use Committee of Wenzhou Medical University in accordance with the Guidelines for the Care and Use of Laboratory Animals.

### 2.2 Immunohistochemical staining

To determine whether CHRNA4 is present on rat Leydig cells, immunohistochemical staining for CHRNA4 in the testis section was performed as described.^20^ 90‐day‐old male rats were used. The testes were fixed in Bouin's solution and embedded in paraffin in the tissue array. 5‐micron‐thick slices were cut. Antigen recovery, peroxidase blocking, CHRNA4 primary antibody (1:250, v/v) binding, HRP‐conjugated secondary antibody ligation, diaminobenzidine staining and Mayer haematoxylin counterstaining were performed. Brown cytoplasmic staining in the cell designates ALC.

### 2.3 Rat ALC isolation

As described previously,^21^ testes from adult rats killed by carbon dioxide asphyxiation were used for ALC isolation. Briefly, testis was infused with collagenase via testicular artery, digested with collagenase and DNase at 34°C. Cells were filtered with 100 μm nylon mesh, followed by Percoll gradient centrifugation and bovine serum albumin gradient centrifugation to collect ALC fraction. ALCs were stained by 3β‐HSD1 histochemical staining to identify the purity as described.^22^ The purity of ALCs was 92%‐95%.

### 2.4 Immunofluorescence staining

Immunofluorescence staining of CHRNA4 was performed as described.^23^ ALCs were isolated from adult rats (90 days old) as above and grown on glass coverslips. Cell fixation with 4% paraformaldehyde, non‐specific antigen blocking with 1% donkey serum, CHRNA4 primary antibody (1:200 dilution) incubation, Alexa‐conjugated anti‐rabbit IgG (1:500) linking and 4′,6‐diamidino‐2‐phenylindole (DAPI) (Beyotime Biotech) counterstaining were performed. The images were taken with a fluorescence microscope and merged.

### 2.5 ALC culture

As previously mentioned,^24^ ALCs were seeded in a 12‐well plate at a cell density of 0.5 × 10^6^ cells/well. After 3 hours, when ALCs were attached to the bottom of well, 1 mL DMEM/F12 (1:1, v/v) together with various concentrations of CAC (0‐50 μmol/L) was added. Cells were cultured at 34°C for 12 hours under 5% CO_2_. Medium and cells were harvested for testosterone and gene expression analysis. To investigate whether CAC affects the cAMP pathway in testosterone synthesis, ALCs were treated with 50 μmol/L CAC without (basal) or LH (10 ng/mL) or 8Br‐AMP (10 mmol/L) for 12 hours. Steroid substrates for testosterone synthesis cascade were added to medium to investigate the effects of CAC on each enzyme catalysis.

### 2.6 MLTC‐1 cell culture

MLTC‐1 cell line is mouse tumour Leydig cell line and can make testosterone.^25^ MLTC‐1 cell line was obtained from Shanghai Zhongqiao Xinzhou Biotech and cultured in RPMI‐1640 medium (Gibco) supplemented with 100 units/mL penicillin and 100 units/mL streptomycin, and 10% (v/v) foetal bovine serum (Gibco). Cells were cultured as above.

### 2.7 Measuring the testosterone concentration in the medium

After 12 hours of treatment, the medium was collected and testosterone was measured as described.^26^ Using the IMMULITE 2000 Total testosterone kit (Siemens), the concentration of testosterone was determined. The minimum detection concentration of testosterone is 0.2 ng/mL.

### 2.8 Measurement of intracellular cAMP level in ALCs

After treatment, ALCs were collected for cAMP measurement by cAMP ELISA kit (Jianglai Bio). In brief, ALCs were scraped off, sonicated and centrifuged at 2800 *g*. Supernatant was collected, ELISA kit was performed, and plate was read for optical density value at 450 nm.

### 2.9 RNA extraction and qPCR

RNA was extracted from ALCs and MTLC‐1 cells using Trizol (Invitrogen), and qPCR was performed as described.^23^ RNA concentration was measured with a NanoDrop 2000 (Thermo). Promega reverse transcription kit was used for preparation of cDNA. The qPCR was performed by SYBR Green detection system (Bio‐Rad) in the Bio‐Rad qPCR system. Ct value was used for the standard curve method to find the level of target mRNA *Lhcgr, Scarb1, Star, Tspo, Cyp11a1, Hsd3b1, Cyp17a1, Hsd17b3* and *Rps16* were detected for rat ALCs. *Lhcgr, Scarb1, Star, Cyp17a1, Hsd3b6, Nr5a1, Nr4a1, Insl3* and *Rps16* were detected for MLTC‐1 cells. The mRNA level was normalized to the housekeeping gene (*Rps16*) level as described.^27^

### 2.10 Western blot

Protein extraction from ALCs and MLTC‐1 cells and Western blot were performed as previously described.^24^ The BCA analysis kit (Beyotime Biotech) was used to measure protein concentration. The following antibodies (LHCGR, STAR, SCARB1, HSD3B1, pERK1/2, ERK1/2, CHRNA4, ACTINB) were used to detect antigens. Blot detection kit (Advansta) was used for Western blotting. The density of the protein was quantified using Image Lab and adjusted to β‐actin (ACTINB).

### 2.11 Statistics

All data are expressed as mean ± SEM. The data were analysed by Student's *t* test to detect significant differences between the two groups. The data were analysed using one‐way analysis of variance and then by Turkey's multiple comparison. All experiments are repeated 3‐5 times. At *P* < .05, the results are considered to be significantly different. GraphPad (GraphPad) was used for analysis.

## 3 RESULTS

### 3.1 Detection of CHRNA4 in rat testis

**Figure 1:**
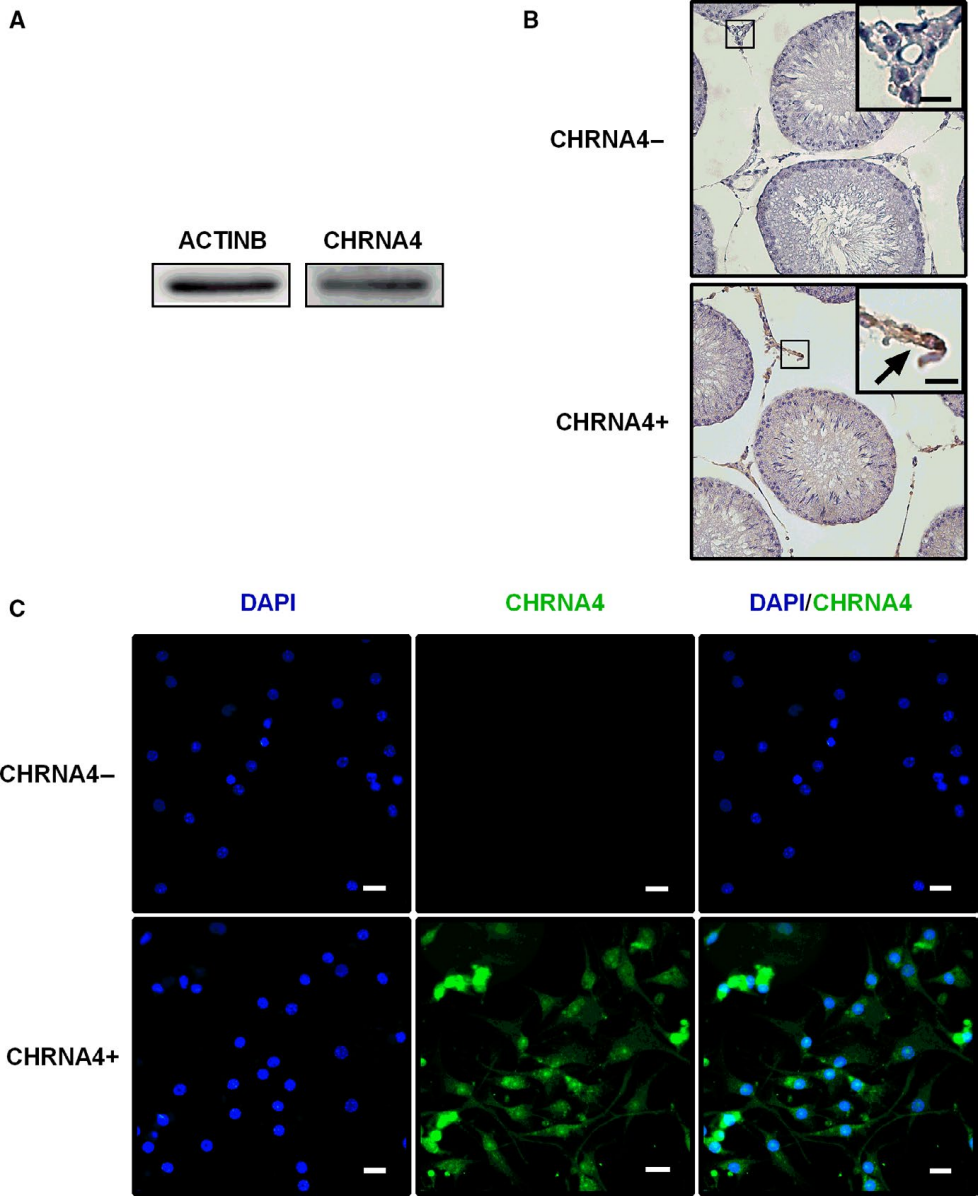
Presence of CHRNA4 on rat adult Leydig cells (ALCs). A, Western blotting band. B, immunohistochemistry of adult rat testis cross‐section. CHRNA4‐ is the CHRNA4‐negative control; CHRNA4+ is the CHRNA4‐positive group; and the brown cells are CHRNA4‐positive ALCs. C, Immunofluorescence. CHRNA4− is the CHRNA4‐negative control; CHRNA4+ is the CHRNA4‐positive ALCs; blue is DAPI; green is CHRNA4; and DAPI/CHRNA4 is merged

First, we analysed the presence of CHRNA4 in adult testes using Western blotting. In fact, CHRNA4 can be detected in adult rat testes (Figure 1A). Then, we performed immunohistochemical staining of CHRNA4 in the testis of adult rats, and we found that CHRNA4 was completely located in the ALCs of the interstitium of rat testis (Figure 1C). Finally, we purified rat ALCs and used them for immunofluorescence analysis of CHRNA4 in cells. We found that the purified rat ALCs were positive for CHRNA4. These data indicate that CHRNA4 is present in the ALC of rat testis.

### 3.2 CAC stimulates testosterone secretion by Leydig cells

**Figure 2:**
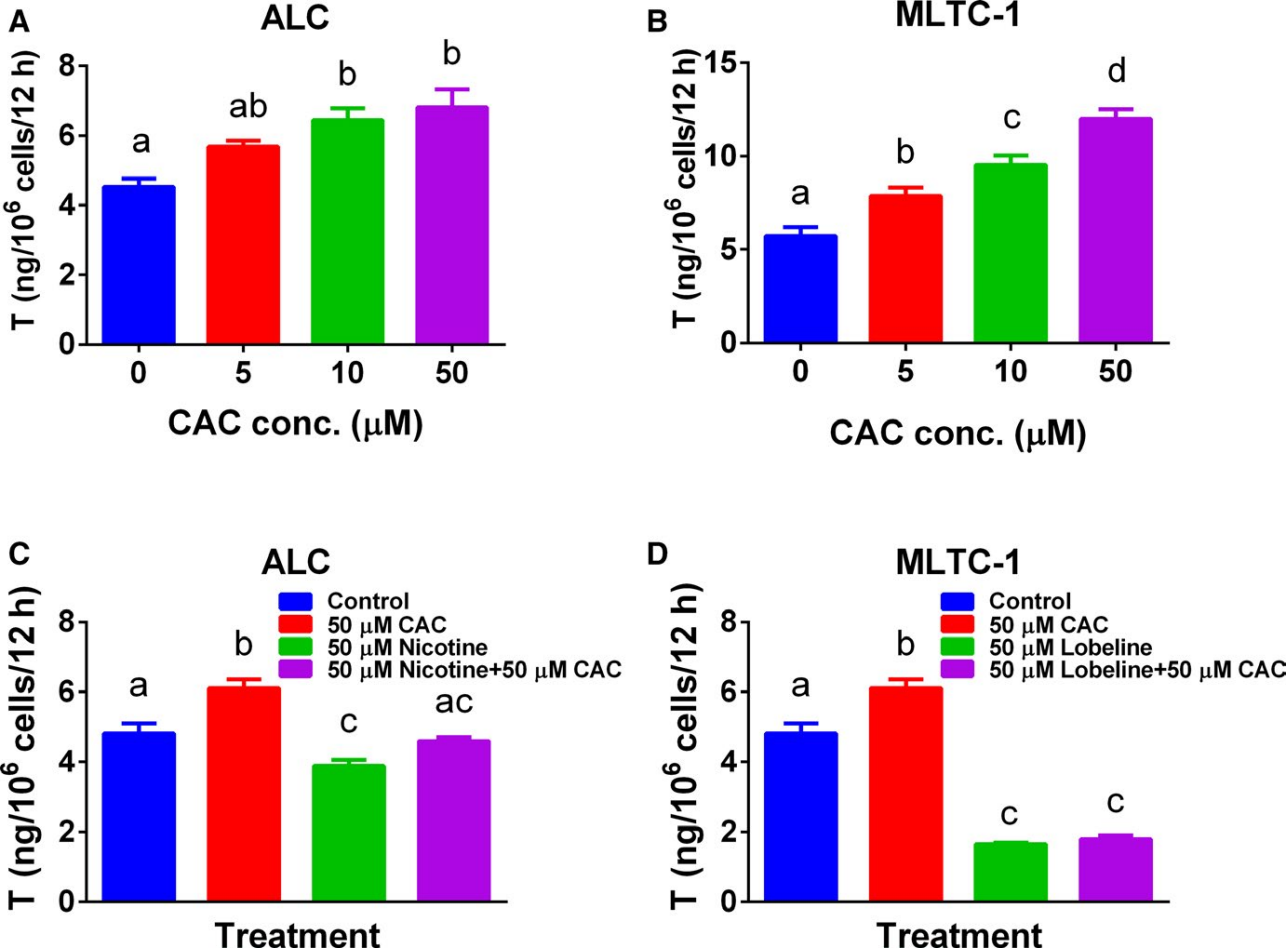
Effect of nAChR agonists (nicotine and lobeline) and antagonist (cisatracurium, CAC) on testosterone (T) secretion in rat adult Leydig cells (ALCs) and MLTC‐1 cells. A, Dose‐dependent increase in the Level of T after 12 h of CAC (5‐50 µmol/L) treatment in rat ALCs; (B) in MLTC‐1 cells; (C) CAC antagonizes nicotine action in ALCs; (D) CAC antagonizes lobeline action in MLTC‐1 cells. Mean ± SEM, n = 4; Similar letter designates no significant difference between two groups at *P* < .05

We treated rat ALCs and mouse MLTC‐1 cells with 0, 5, 10 and 50 μmol/L CAC for 12 hours. CAC dose‐dependently increased testosterone levels in rat ALCs, with statistical significance recorded at 10 and 50 μmol/L (Figure 2A). CAC also significantly stimulated testosterone production in mouse MLTC‐1 cells at ≥5 μmol/L (Figure 2B). These data indicate that CAC is a stimulant of testosterone production.

### 3.3 CAC stimulates testosterone production in rat ALCs through nAChR

To determine the mechanism of CAC, we used nAChR agonists (nicotine and lobeline). Compared with the control group, the nAChR agonists nicotine (Figure 2C) and lobeline (Figure 2D) significantly inhibited the testosterone levels secreted by ALCs, whereas CAC stimulated the testosterone output secreted by ALCs. However, the combination of CAC with nicotine (Figure 2C) and lobeline (Figure 2D) reversed the CAC‐mediated stimulation. These data indicate that CAC works through nAChR.

### 3.4 CAC increases intracellular cAMP levels in rat ALCs

**Figure 3:**
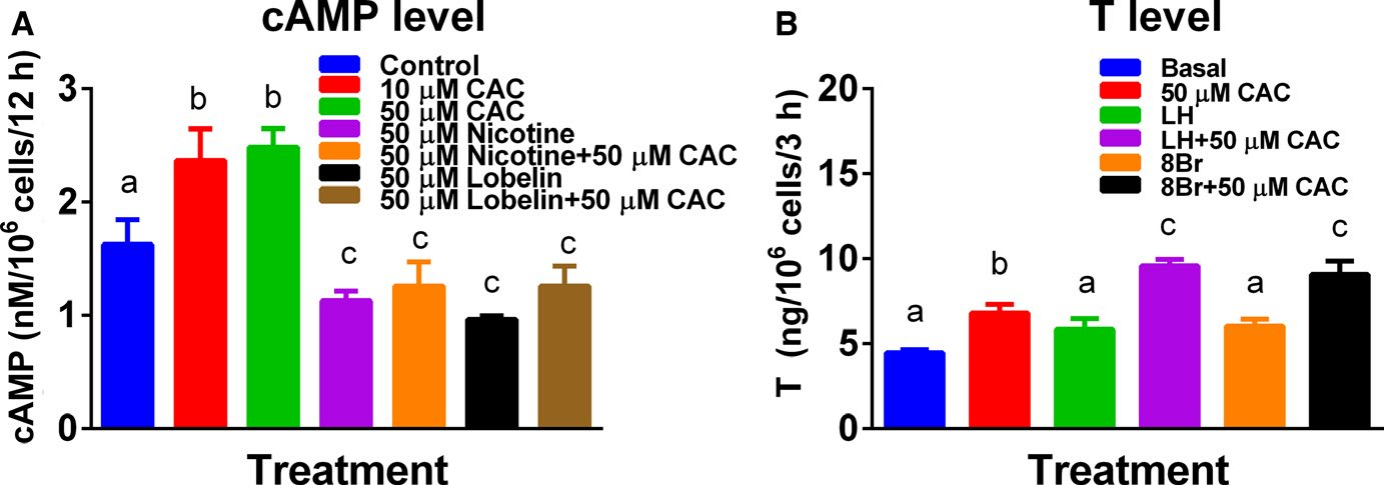
Cisatracurium (CAC) enhances cAMP levels and basal, LH‐ and cAMP‐stimulated testosterone (T) production in adult Leydig cells (ALCs). A, cAMP levels after 12 h treatment; (B) T levels under basal condition, LH stimulation or 8Br‐cAMP stimulation. All groups in panel were cultured for 3 h. Mean ± SEM, n = 4. Similar letter designates no significant difference between two groups at *P* < .05

Cisatracurium significantly increased intracellular cAMP levels in rat ALCs (Figure 3A), whereas treatment with nAChR agonists (nicotine and lobeline) significantly reduced intracellular cAMP levels (Figure 3A). In this cell type, previous observation confirmed that nicotine reduced cAMP levels.^12^ However, the combination of CAC with nicotine and lobeline reversed the CAC‐mediated stimulation of cAMP production (Figure 3A). These data indicate that CAC acts through nAChR to increase cAMP levels.

### 3.5 CAC increases testosterone secretion by ALCs under LH and 8Br‐cAMP stimulation

In addition to the aforementioned CAC stimulation of testosterone production in ALCs under basal condition, we also studied testosterone output under 3h‐incubated basal, LH and 8Br‐cAMP‐stimulated conditions after ALCs were treated with CAC for 12 hours. In fact, under the stimulation of basal, LH and 8Br‐cAMP, CAC significantly increased testosterone output at 50 μmol/L (Figure 3B). This indicates that CAC acts on the downstream cascade of cAMP.

### 3.6 CAC enhances testosterone production in rat ALCs mediated by pregnenolone

**Figure 4:**
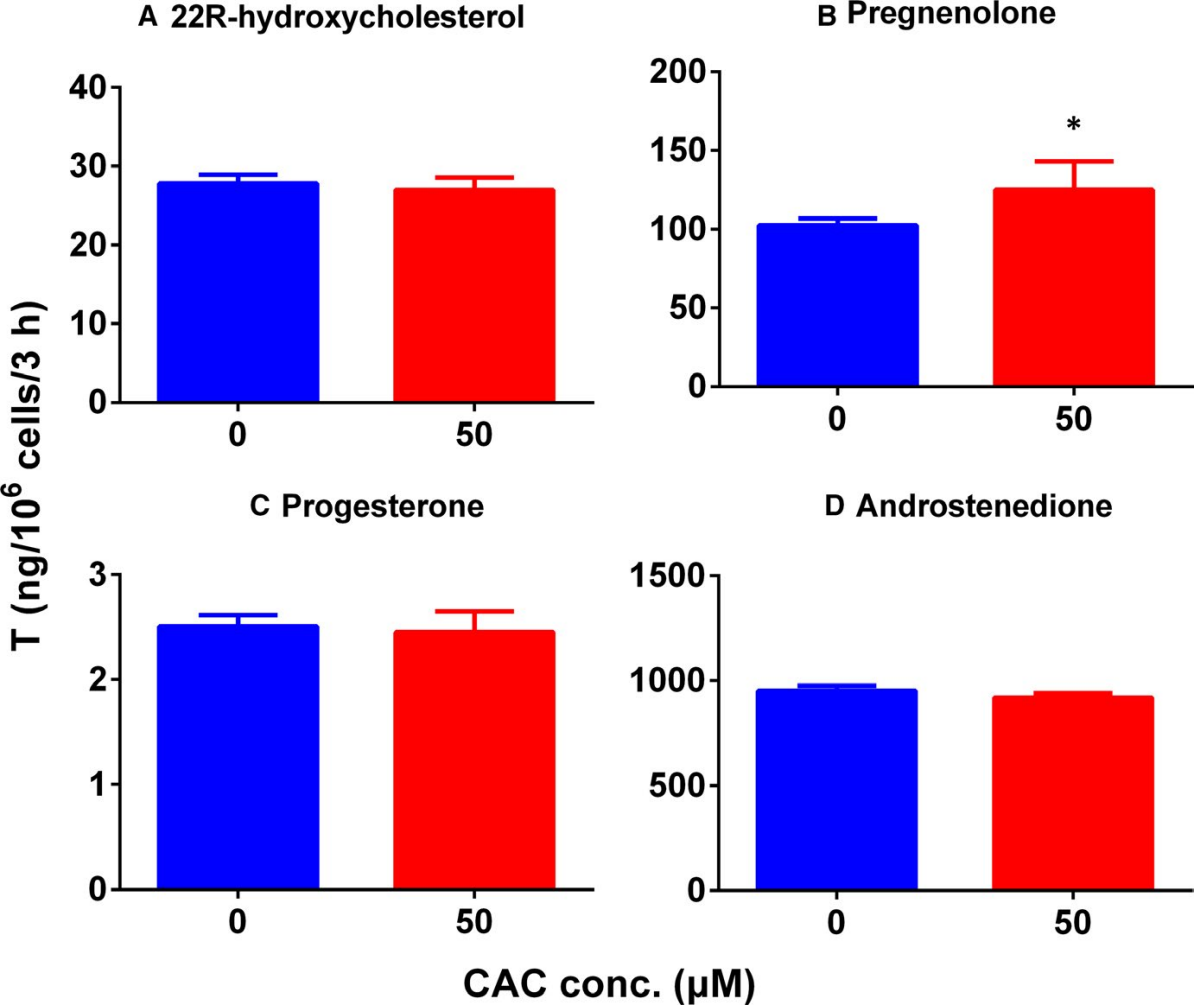
Effect of cisatracurium (CAC) on testosterone synthesis when different substrates are added in T biosynthetic pathway. A, 22R‐hydroxychlesterol; (B) pregnenolone; (C) progesterone; (D) androstenedione. All groups were cultured for 3 h. Mean ± SEM, n = 4. * indicates significant difference when compared to control at *P* < .05

To check whether CAC also acts on any of the enzyme activity of testosterone biosynthetic pathway, we added various substrates, including 22R‐hydroxycholesterol of CYP11A1, pregnenolone of 3β‐HSD1, progesterone of CYP17A1 and androstenedione of 17β‐HSD3. We found that CAC enhanced testosterone production in rat ALCs only when pregnenolone was added as a substrate (Figure 4).

### 3.7 CAC up‐regulates the expression of steroidogenesis‐related genes in rat ALCs

**Figure 5:**
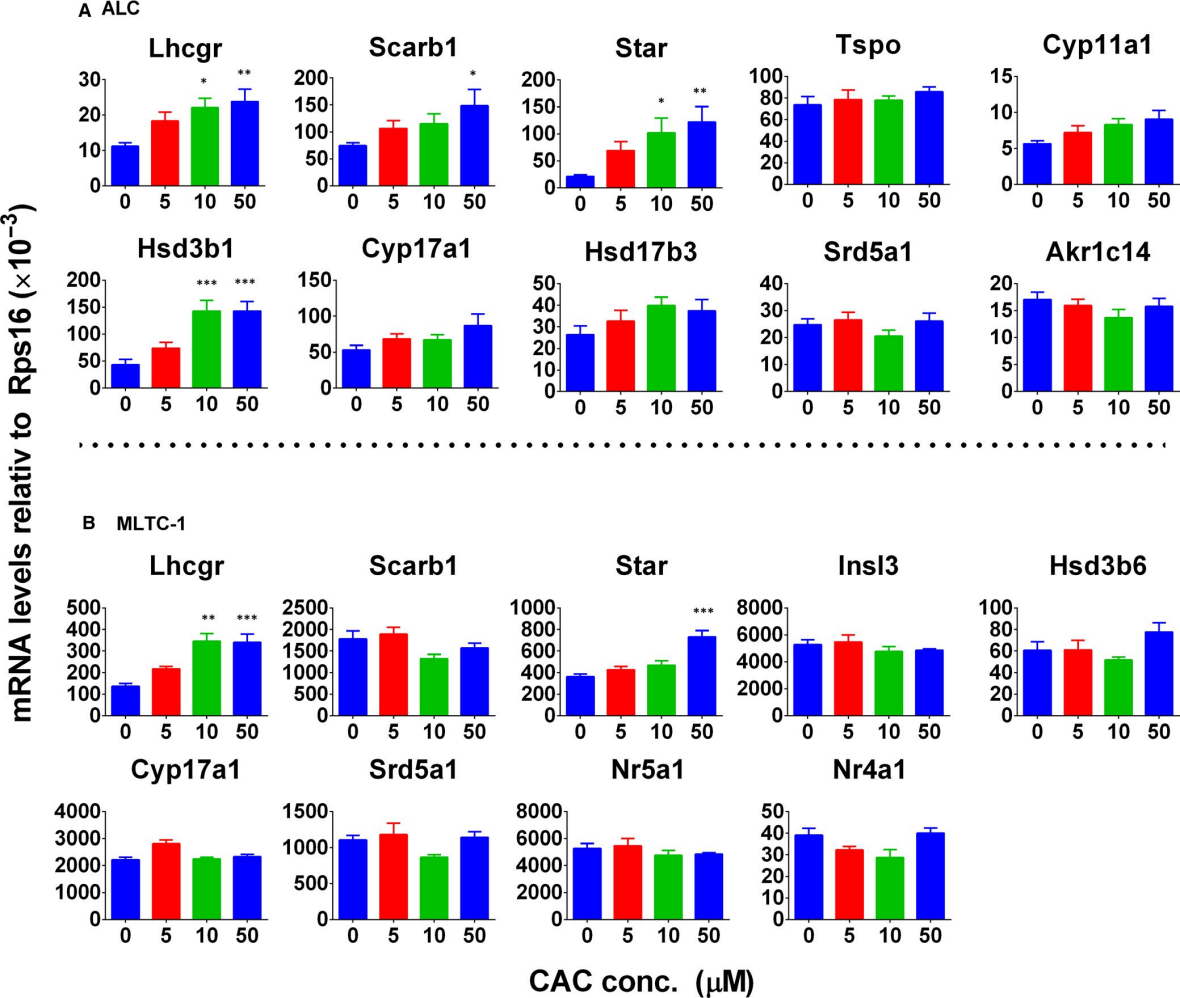
The effect of cisatracurium (CAC) on up‐regulation of steroidogenesis‐related gene expression in adult Leydig cells (ALCs) and MLTC‐1 cells. Rat ALCs or MLTC‐1 cells were cultured with 0‐50 μmol/L CAC for 12 h. The expression of Leydig cell genes was measured by qPCR and calculated relative to *Rps16*, the internal control. A, ALC genes; (B) MLTC‐1 genes. Mean ± SEM, n = 5. Asterisks (*, **, ***) designate significant differences from the control at *P* < .05, 0.01 and 0.001, respectively

We examined the gene expression in the steroidogenic pathway of ALCs in rats after 12 hours of CAC treatment. We found that CAC significantly up‐regulated the expression of *Lhcgr*, *Star,* and *Hsd3b1* at 10 and 50 μmol/L (Figure 5A), and significantly up‐regulated the expression of *Scarb1* at 50 μmol/L (Figure 5A). This indicates that CAC up‐regulates the expression of several genes in the steroidogenic cascade in rat ALCs. CAC did not affect the expression of other genes (*Tspo*, *Cyp11a1*, *Cyp17a1*, *Hsd17b3*, *Srd5a1* and *Akr1c14*).

### 3.8 CAC up‐regulates the expression of steroidogenesis‐related genes in mouse MLTC‐1 cells

We also examined the gene expression of mouse MLTC‐1 cells in the steroidogenic cascade after 12 hours of CAC treatment. We found that CAC significantly up‐regulated *Lhcgr* expression at 10 and 50 μmol/L, and significantly increased *Star* expression at 50 μmol/L (Figure 5B). Interestingly, we did not find any changes in *Scarb1, Hsd3b6, Cyp17a1* and *Srd5a1* (Figure 5B). We also examined the expression of transcription factors (*Nr5a1* and *Nr4a1*) and the peptide INSL3 (*Insl3*) in MLTC‐1 cells. It did not change their expressions.

### 3.9 CAC increases protein levels in rat ALCs and mouse MLTC‐1 cells

**Figure 6:**
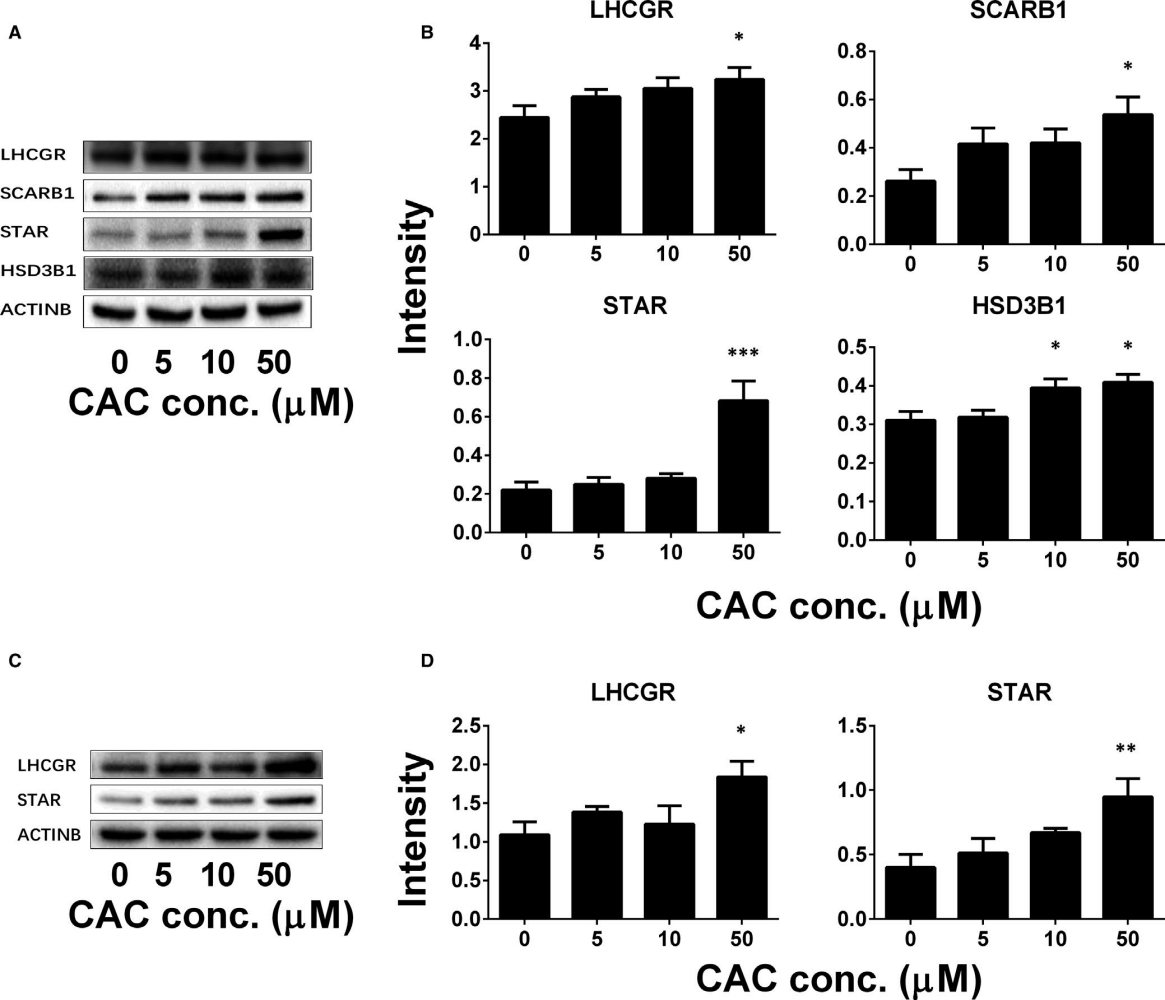
The effect of cisatracurium (CAC) on the increase of protein levels in adult Leydig cells (ALCs) and MLTC‐1 cells. Rat ALCs and mouse MLTC‐1 cells were cultured with 0‐50 µmol/L CAC for 12 h. The levels of LHCGR, SCARB1, STAR and HSD3B1 were measured by Western blotting and adjusted relatively to β‐actin (ACTINB), the internal control. A, B, Western blot image and quantitative data, respectively, for ALCs; (C, D) Western blot image and quantitative data, respectively, for MLTC‐1 cells. Mean ± SEM, n = 4. Asterisks (*, **, ***) designate significant differences when compared to control at *P* < .05, 0.01 and 0.001, respectively

We further examined the protein levels of LHCGR, SCARB1, STAR and HSD3B1 in rat ALCs (Figure 6A,B). In fact, CAC significantly increased LHCGR, SCARB1, STAR and HSD3B1 in ALCs at concentrations of 10 and/or 50 μmol/L (Figure 6B). We also examined the protein levels of LHCGR and STAR in mouse MLTC‐1 cells (Figure 6C,D) and found that CAC significantly increased LHCGR and STAR levels at 50 μmol/L. These results indicate that after CAC treatment, changes in protein levels and their mRNA levels are parallel.

### 3.10 CAC enhances phosphorylation of ERK1/2

**Figure 7:**
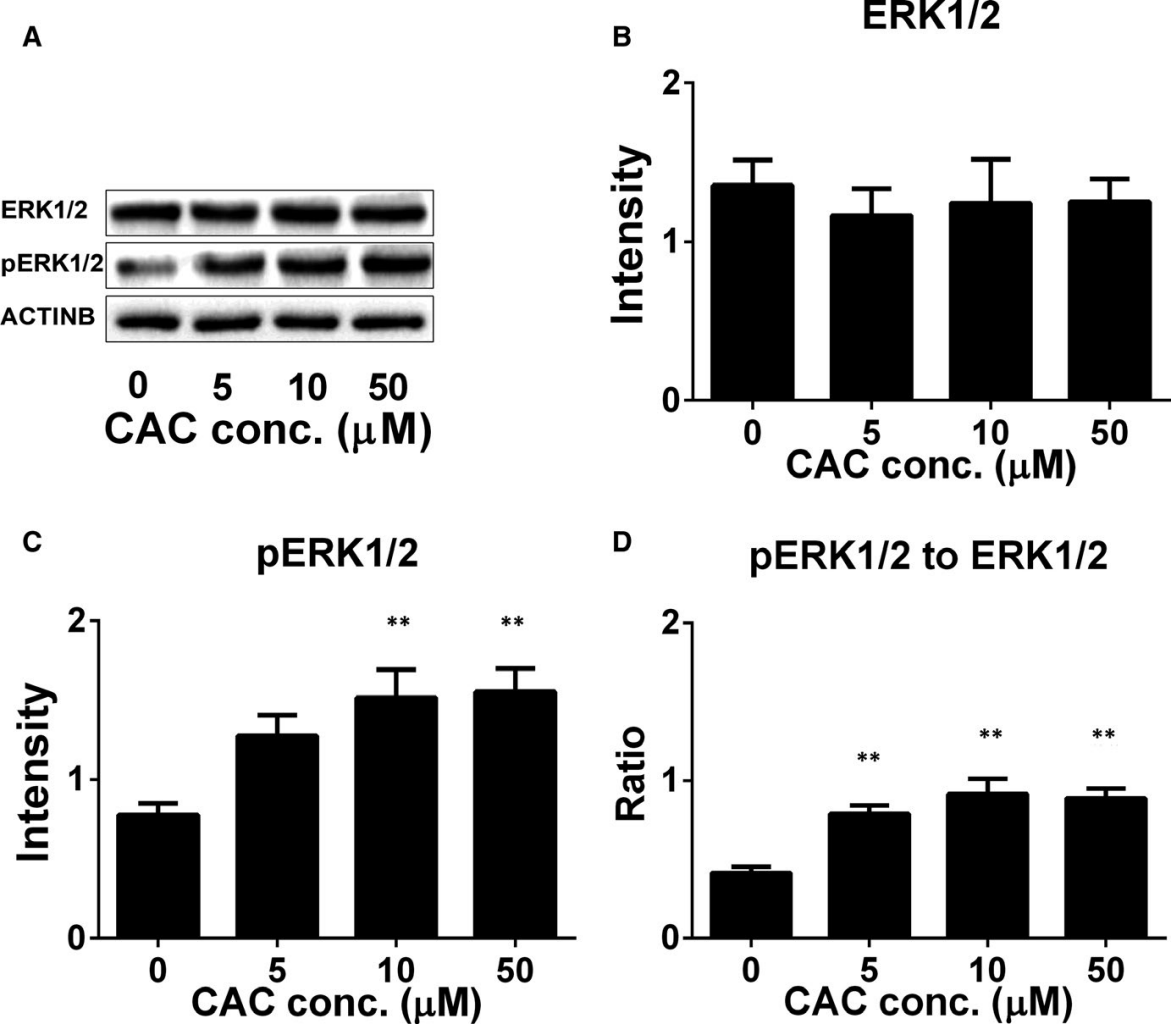
The effect of cisatracurium (CAC) on ERK1/2 phosphorylation. Rat adult Leydig cells (ALCs) were cultured with 0‐50 μmol/L CAC for 12 h. The expression of pERK1/2 and ERK1/2 was measured by Western blot and calculated relatively to ACTINB, the internal control. A, Western blot band; (B) total ERK1/2 levels; (C) pERK1/2 levels; (D) ratio of pERK1/2 to ERK1/2. Mean ± SEM, n = 4. Asterisk (**) designates significant difference from the control at *P* < .01

Previous studies have shown that LHCGR activation in Leydig cells can also increase ERK1/2 phosphorylation signalling in addition to the cAMP/PKA pathway. ^28^, ^29^ Because LHCGR was significantly up‐regulated, we examined the ERK1/2 signalling pathway after CAC treatment. In fact, CAC increased ERK1/2 phosphorylation without affecting total ERK1/2 levels (Figure 7), indicating that the ERK1/2 pathway is also involved in CAC‐mediated testosterone synthesis.

## 4 DISCUSSION

The current study clearly demonstrated that nAChR subunit CHRNA4 is exclusively expressed in Leydig cells of rat testis. Early in vitro studies have shown that nicotine as an nAChR agonist can inhibit testosterone synthesis in Leydig cells.^12^, ^30^ In recent years, some in vivo studies have also demonstrated the inhibitory effect of nicotine in the synthesis of testosterone by Leydig cells. Nicotine administration (0.5 or 1 mg/kg/d) to pubertal rats for 2 weeks can significantly reduce serum testosterone levels by lowering the number of Leydig cells and down‐regulating the expression of steroidogenic gene expression (including *Hsd3b1*).^31^ Prenatal nicotine exposure also led to the inhibition of testosterone synthesis by Leydig cells in the offspring.^32^ The expression of *Star* and *Hsd3b1* was significantly down‐regulated in the testis of progeny after nicotine exposure and in MLTC‐1 cells after in vitro nicotine treatment.^32^ The current study investigated the effects of CAC, a nAChR antagonist, on Leydig cell function. After CAC treatment, the expression of *Star* and *Hsd3b1* was significantly up‐regulated (Figure 5) and testosterone levels were significantly increased (Figure 2). At the same time, CAC can antagonize nicotine‐mediated inhibition on testosterone synthesis (Figure 2C). As described in previous studies,^33^, ^34^ the secretion of androgen in Leydig cells requires a LHCGR signalling, which depends on the activation of intracellular cAMP. However, nAChR activation can inhibit LH/hCG‐stimulated testosterone production in the testis by lowering cAMP levels.^35^ Here, as an antagonist of nAChR, CAC bound to nAChR to reverse the inhibitory effect on cAMP production by its agonist nicotine or lobeline, thus resulting in increased testosterone levels.

Cisatracurium has many advantages, so it has been widely used in clinic. In this study, we also provide a novel benefit of CAC, which can increase testosterone synthesis and can protect Leydig cells from adverse effects by nicotine.

Cisatracurium significantly increased testosterone synthesis in both rat ALCs and mouse MLTC‐1 cell line after 12 hours of treatment. The mechanism of stimulating testosterone synthesis in ALCs and MLTC‐1 cells by CAC seems similar. We found that in ALCs and MLTC‐1 cells, the expression of *Lhcgr* and *Star* and their proteins was up‐regulated by CAC. The rate‐limiting step for testosterone synthesis is the transport of cholesterol from the cytoplasm into the inner membrane of the mitochondrion, which is assisted by STAR protein.^36^ Knockout of STAR caused almost no steroid production in the testis and the adrenal gland.^37^ Upon the activation of LHCGR after LH stimulation, adenylate cyclase is activated and intracellular cAMP levels are increased, thus inducing the expression of STAR and its phosphorylation, leading to an increase in the rate of cholesterol transport to the mitochondrion.^38^, ^39^ Therefore, CAC‐mediated increase the synthesis of testosterone in ALCs and MLTC‐1 cells by accelerating the transport of cholesterol to the mitochondrion.

There are other cascades involved in promoting testosterone synthesis in ALCs and MLTC‐1 cells. In ALCs, after CAC treatment, the expression of *Scarb1* was up‐regulated. *Scarb1* encodes a membrane receptor, SCARB1, which provides active uptake of cholesterol from circulation via endocytosis of high‐density lipoprotein.^40^ Therefore, increasing SCARB1 expression results in increased levels of intracellular cholesterol, leading to the increased testosterone synthesis. However, SCARB1 was not up‐regulated in MLTC‐1 cells by CAC, suggesting that there is a different regulation, depending on cell types. Besides, *Hsd3b1* was also up‐regulated in rat ALCs by CAC. 3β‐HSD1, encoded by *Hsd3b1*, is an smooth endoplasmic reticulum enzyme that is responsible for catalysing pregnenolone to progesterone in rat ALCs.^14^ Indeed, CAC significantly increased the conversion of pregnenolone to progesterone, leading to the increase of testosterone levels, at 50 μmol/L. The homolog of rat 3β‐HSD1 in ALCs in 3β‐HSD6 (encoded by *Hsd3b6*) in mouse MLTC‐1 Leydig cells.^41^ Apparently, *Hsd3b6* was not affected in MLTC‐1 cells after CAC treatment.

Although the primary signalling pathway of LHCGR is cAMP/PKA signalling, other signalling is also involved. The ERK1/2 signalling pathway is related to testosterone synthesis in Leydig cells.^42^ There is a cross‐talk between cAMP/PKA and ERK1/2 pathways. Indeed, it has been reported that LH activated the ERK1/2 signalling partially via PKA signalling.^43^ However, PKA‐independent activation of ERK1/2 is also involved after LH stimulation, which involves the release of soluble factors that act to phosphorylate the epidermal growth factor receptor in an autocrine/paracrine fashion.^44^ Here, we found that CAC increased phosphorylation of EKR1/2, which may lead to increased testosterone production in ALCs.

As a muscle relaxant and anaesthetic, CAC is widely used for general anaesthesia and respiratory management. The benefit of CAC to increase testosterone synthesis may widen CAC application, such as to antagonize nicotine‐mediated side effects.

In conclusion, CAC significantly increased testosterone synthesis by enhancing cholesterol transport to cells (via SCARB1) and mitochondrion (via STAR) as well as the up‐regulated expression of LHCGR and 3β‐HSD1. CAC may bind to CHNR4A1 to cause the activation of cAMP and ERK1/2 phosphorylation for signalling.

## CONFLICT OF INTEREST

The authors declare that they have no conflict of interest to declare.

## AUTHOR CONTRIBUTIONS

**Chaobo Ni:** Investigation (lead); Methodology (lead); Writing‐original draft (equal). **Yang Li:** Data curation (equal); Methodology (equal). **Zengqiang Li:** Formal analysis (equal); Investigation (equal). **Lili Tian:** Data curation (supporting); Methodology (supporting). **Jie Fu:** Data curation (supporting); Methodology (supporting). **Keyang Wu:** Methodology (supporting). **Yiyan Wang:** Project administration (supporting); Supervision (supporting). **Ming Yao:** Conceptualization (equal); Writing‐original draft (supporting). **Renshan Ge:** Project administration (lead); Supervision (lead); Writing‐original draft (equal).

## Supporting information

Table S1‐3

## Data Availability

The data that support the findings of this study are available from the corresponding author upon reasonable request.
