# Supplementary material for: Cisatracurium stimulates testosterone synthesis in rat and mouse Leydig cells via nicotinic acetylcholine receptor
Source: J Cell Mol Med. 2020 Oct 27;24(24):14184–94. doi: 10.1111/jcmm.16029 (PMC7754058; doi:10.1111/jcmm.16029)
Supplement: Supplementary file 1 — Table S1‐3 [file JCMM-24-14184-s001.docx]

**Supplementary Table S1. Primer information in rats**

| **Primer**  **Symbol** | **Gene name** | **Primer direction** | **Sequences (5’to 3’)** | **PCR**  **(bp)** | **Accession** |
| --- | --- | --- | --- | --- | --- |
| Lhcgr | Luteinizing hormone receptor | Forward | CTGCGCTGTCCTGGCC | 103 | NM_012978 |
|  |  | Reverse | CGACCTCATTAAGTCCCCTGAA |  |  |
| Scarb1 | Scavenger receptor class B, member 1 | Forward | ATGGTACTGCCGGGCAGAT | 117 | NM_031541 |
|  |  | Reverse | CGAACACCCTTGATTCCTGGTA |  |  |
| Star | Steroidogenic acute regulatory protein | Forward | CCCAAATGTCAAGGAAATCA | 187 | NM_031558 |
|  |  | Reverse | AGGCATCTCCCCAAAGTG |  |  |
| Tspo | Translocator protein | Forward | GGTGGACCTCATGCTTGTCA | 162 | NM_012515.2 |
|  |  | Reverse | CCTCGCCGACCAGAGTTATC |  |  |
| Cyp11a1 | Cholesterol side chain cleavage enzyme | Forward | AAGTATCCGTGATGTGGG | 127 | NM_017286 |
|  |  | Reverse | TCATACAGTGTCGCCTTTTCT |  |  |
| Hsd3b1 | 3β-Hydroxysteroid dehydrogenase 1 | Forward | CCCTGCTCTACTGGCTTGC | 189 | NM_001007719 |
|  |  | Reverse | TCTGCTTGGCTTCCTCCC |  |  |
| Cyp17a1 | 17α-hydroxylase/ 17,20-lyase | Forward | TGGCTTTCCTGGTGCACAATC | 90 | NM_012753 |
|  |  | Reverse | TGAAAGTTGGTGTTCGGCTGAAG |  |  |
| Hsd17b3 | 17β-Hydroxysteroid dehydrogenase 3 | Forward | TGAAAGTTGGTGTTCGGCTGAAG | 202 | NM_054007 |
|  |  | Reverse | TGAAAGTTGGTGTTCGGCTGAAG |  |  |
| Srd5a1 | Steroid 5α-reductase 1 | Forward | CAATCCTGCAAGATTCCACC | 206 | J05035 |
|  |  | Reverse | ATTGGTCCTTGGGTGCATTC |  |  |
| Akr1c14 | 3α-Hydroxysteroid dehydrogenase | Forward | GCAGCGTGGGGTTGTG | 172 | NM_138547 |
|  |  | Reverse | TGGATGATTGGGATGGTCA |  |  |
| Rps16 | Ribosomal protein S16 | Forward | AAGTCTTCGGACGCAAGAAA | 148 | [NM_001169146](https://www.ncbi.nlm.nih.gov/entrez/viewer.fcgi?db=nucleotide&id=310703681) |
|  |  | Reverse | TTGCCCAGAAGCAGAACAG |  |  |

**Supplementary Table S2. Primer information in mouse MLTC-1 cells**

| **Primer**  **Symbol** | **Gene name** | **Primer direction** | **Sequences (5’to 3’)** | **PCR**  **(bp)** | **Accession** |
| --- | --- | --- | --- | --- | --- |
| Lhcgr | Luteinizing hormone receptor | Forward | AGAGTGATTCCCTGGAAAGGA | 103 | NM_001364898.1 |
|  |  | Reverse | TCATCCCTTGGAAAGCATTC |  |  |
| Scarb1 | Scavenger receptor class B, member 1 | Forward | GCCAGCGTGCTTTTATGA | 117 | NM_001205082.1 |
|  |  | Reverse | CCGTTCCATTTGTCCACC |  |  |
| Star | Steroidogenic acute regulatory protein | Forward | TGTCAAGGAGATCAAGGTCCTG | 187 | NM_011485.5 |
|  |  | Reverse | CGATAGGACCTGGTTGATGAT |  |  |
| Cyp17a1 | Cholesterol side chain cleavage enzyme | Forward | CCAGGACCCAAGTGTGTTCT | 127 | NM_007809.3 |
|  |  | Reverse | CCTGATACGAAGCACTTCTCG |  |  |
| Hsd3b6 | 3 beta-hydroxysteroid dehydrogenase/Delta 5-->4-isomerase type 6 | Forward | GGAGGAGATCAGGGTCCTGG | 189 | NM_013821.3 |
|  |  | Reverse | CTAGGATGGTCTGCCTGGG |  |  |
| Srd5a1 | Steroid 5α-reductase 1 | Forward | CAATCCTGCAAGATTCCACC | 206 | J05035 |
|  |  | Reverse | ATTGGTCCTTGGGTGCATTC |  |  |
| Nr5a1 | nuclear receptor subfamily 5 group A member 1 | Forward | AGGTGTCGGGCTACCACTAC | 90 | NM_001316687.1 |
|  |  | Reverse | CCACCCCGCATTCGATCAG |  |  |
| Nr4a1 | nuclear receptor subfamily 4 group A member 1 | Forward | TTGAGTTCGGCAAGCCTACC | 202 | XM_030248339.1 |
|  |  | Reverse | GTGTACCCGTCCATGAAGGTG |  |  |
| Insl3 | insulin like 3 | Forward | CGCTGCTACTGATGCTCCT | 172 | NM_013564.7 |
|  |  | Reverse | ACAGGTCTTGCTGGGTGC |  |  |
| Rps16 | Ribosomal protein S16 | Forward | TGAAGGGTGGTGGACATG | 148 | NM_013647.2 |
|  |  | Reverse | AATAAGCTACCAGGGCCTT |  |  |

**Supplementary Table S3. Antibodies**

| **Antibody** | **Species** | **Vendor (City, State, catalogue)** | **Dilution** | | |
| --- | --- | --- | --- | --- | --- |
|  |  |  | **WB** | **IHC-P** | **IF** |
| ACTINB | rabbit | Cell Signaling Technology (Danvers, MA) | 1:1000 | - | - |
| CHRNA4 | rabbit | Abcam (San Francisco, CA) | 1:800 | 1:250 | 1:200 |
| LHCGR | rabbit | Multi Sciences (Hangzhou, China) | 1:1000 | - | - |
| SCARB1 | rabbit | Multi Sciences (Hangzhou, China) | 1:1000 | - | - |
| STAR | mouse | Cell Signaling Technology (Danvers, MA) | 1:500 | - | - |
| HSD3B1 | mouse | Novus Biologicals (Littleton, CO) | 1:1000 | - | - |
| ERK1/2 | mouse | Abcam (San Francisco, CA) | 1:1000 | - | - |
| pERK1/2 | mouse | Abcam (San Francisco, CA) | 1:10000 | - | - |
| HRP-conjugated anti-rabbit IgG | goat | Multi Sciences (Hangzhou, China) | 1:2000 | - | - |
| HRP-conjugated anti-mouse IgG | goat | Multi Sciences (Hangzhou, China) | 1:2000 | - | - |
| Alexa488-conjugated anti-rabbit IgG | goat | Abcam (San Francisco, CA) | - | - | 1:500 |

WB = Western blot.
